# Evaluation of pharmacological activities and assessment of intraocular penetration of an ayurvedic polyherbal eye drop (Itone™) in experimental models

**DOI:** 10.1186/1472-6882-13-1

**Published:** 2013-01-02

**Authors:** Thirumurthy Velpandian, Pankaj Gupta, Alok Kumar Ravi, Hanuman Prasad Sharma, Nihar Ranjan Biswas

**Affiliations:** 1 Department of Ocular Pharmacology and Pharmacy, Dr. Rajendra Prasad Centre for Ophthalmic Sciences, All India Institute of Medical Sciences, Room No: 634, 6th floor, New Delhi 110029, India; 2 Department of Pharmacology, All India Institute of Medical Sciences, New Delhi 110029, India

**Keywords:** Polyherbal, Antiangiogenic, Anti-inflammatory, Anticataract, Antioxidant, Cytotoxicity, LC-MS/MS

## Abstract

**Background:**

The polyherbal eye drop (Itone™) is a mixture of aqueous distillates of nineteen traditionally used ingredients that sum up to impart potency to the formulation and make it a useful adjunct in various ocular pathologies. However, as there have been no controlled experimental studies accounting to the above claim, therefore, the present study was designed to evaluate the polyherbal formulation (PHF) for antiangiogenic, anti-inflammatory, anticataract, antioxidant and cytotoxicity in addition to the evaluation of intraocular penetration of PHF in rabbit eyes using LC-MS/MS.

**Materials and methods:**

Antiangiogenic activity of the PHF was evaluated using *in ovo* chick chorio-allantoic membrane (CAM) assay and *in vivo* cautery induced corneal neovascularization assay in rats. Anticataract potential was evaluated using steroid induced cataract in developing chick embryos, sodium selenite induced cataract in rat pups and galactose induced cataract in rats. The antioxidant activity was evaluated using di-phenyl picryl hydrazyl (DPPH) radical scavenging assay. Anti-inflammatory activity was evaluated *in vitro* using inhibition of LTB_4_ formation in human WBCs and *in vivo* using carrageenan induced paw edema assay in rats. The cytotoxicity was evaluated against HeLa cancer cell lines using (3-(4,5-Dimethylthiazol-2-yl)-2,5-diphenyltetrazolium bromide (MTT) assay. Furthermore evaluation of the intraocular penetration of the PHF was carried out in rabbit eyes via aqueous humor paracentesis and further analysis using LC-MS/MS.

**Results:**

PHF significantly inhibited VEGF induced proliferation of new blood vessels in CAM assay and inhibited the cautery induced corneal neovascularization in rats. Additionally, PHF showed noticeable delay in the progression of cataract in the selenite and galactose induced cataract models whereby the PHF treated lenses were graded for stages II and III respectively. However, the PHF did not show any anticataract activity in the hydrocortisone induced cataract model. Moreover, PHF exhibited anti-inflammatory activity whereby it showed 39.34% inhibition of LTB_4_ formation and significantly inhibited carrageenan induced paw edema in rats. Eight compounds of PHF viz. camphor, casticin, curcumin-II, quercetin, rosmarinic acid, γ-terpinene, β-pinene and dipentene exhibited transcorneal penetration in rabbit eyes.

**Conclusion:**

The significant antiangiogenic and anti-inflammatory activities evinced by the PHF merits further investigation for ocular neovascular and inflammatory diseases in humans.

## Background

The extracts of herbal products and Ayurvedic, Siddha and Unani formulations are mixtures of at least partially uncharacterized constituents. It is claimed that such a mixture provides a therapeutic advantage, since the unknown constituents may be additive or synergistic in action or may produce a balance to counteract adverse effects of any one constituent. This may provide more efficacy than the known constituent used alone. Thus, purification of the medicines is not required as it may lead to loss of the advantages provided by the mixture [1]. An ayurvedic proprietary polyherbal eye drop (Itone™) developed on the basis of ayurvedic principles have been available for the last 25 years in Indian Market and has been recognized to have medicinal value for various ocular ailments. The polyherbal formulation (Itone™) consists of a fixed combination of the aqueous distillates of nineteen traditionally used ingredients including *Azadirachta indica* (nimba-5%), *Moringa pterygosperma* (sobhanjana-5%), *Eclipta alba* (bhringaraj-5%), *Boerhaavia diffusa* (punarnava-7.5%), *Carum copticum* (yamani-2%), *Terminalia chebula* (haritaki-5%)*, Terminalia belerica* (vibhitaka-5%), *Emblica officinalis* (dhatriphala-5%), *Santalum album* (swet chandan-5%)*,* mukta (pearl-1%), *Ocimum sanctum* (tulsi patra-5%), *Vitex negundo* (nirgundi-5%), *Curcuma longa* (haridra-5%), *Mentha piperata* (menthol-2%), *Cinnamomum camphora* (camphor-3%), *Amomum subulatum* (ela-5%), *Rosa centifolia* (satapatri-7.5%), saindhava laban (rock salt-2%) and madhu (honey-10%). It has been reported to be a useful adjunct in various ocular diseases like conjunctivitis, trachoma, blepharitis, keratitis, corneal ulcers etc. It has been reported to maintain the visual acuity and helpful in computer vision syndrome [2,3].

Several clinical studies are available appreciating the effect of Itone™ in various ocular pathologies. However, controlled experimental studies are not available to substantiate the claim. Therefore, the present study was carried out to systematically evaluate the above polyherbal formulation (PHF) for several ocular indications using contemporary technologies and experimental models to explore antiangiogenic, anticataract, anti-inflammatory, cytotoxicity and antioxidant properties. The above PHF was also subjected for the evaluation of its intraocular penetration after topical application in rabbits. These approaches are expected to increase the modern application of traditional knowledge for their scientific rationality and therapeutic application.

## Methods

### Chemical and reagents

Vascular Endothelial Growth Factor (VEGF) and MTT were purchased from Sigma-Aldrich, USA. Dulbecco’s Modified Eagle Medium (DMEM) was purchased from Invitrogen, India. Hydrocortisone sodium succinate injection was purchased from Alkem laboratories Ltd., Mumbai, India. Sodium selenite was purchased from Loba chemie, India. Riboflavin was purchased from Sisco Research Laboratories Pvt. Ltd., Mumbai, India. Galactose was purchased from Spectrochem Pvt. Ltd., Mumbai, India. The preservative free PHF (Itone™ eye drops, Batch No - 2142) were provided by Dey’s Medical Stores, Kolkata, India. All the other reagents were of analytical grade and were used without further purification.

All study protocols were approved by standing Institutional Animal Ethics Committee (IAEC) of All India Institute of Medical Sciences (AIIMS), New Delhi, India. All animal experiments were done in accordance with guidelines of Association for Research in Vision and Ophthalmology (ARVO).

### Evaluation of antiangiogenic activity of the PHF

#### Chick Chorio-Allantoic Membrane (CAM) assay

The CAM assay was performed as per the procedure described by Ribatti *et al.,* 1997 [4] with slight modifications. Briefly, fresh fertile *white leghorn* chick eggs were purchased from Kegg Farm (Gurgaon, Haryana, India) and further placed in a humidified incubator at 36 ± 2°C. On the third day of incubation, a hole was drilled on the egg shell and 2–3 ml of albumin was withdrawn aseptically. The hole was sealed using sterile parafilm and eggs were re-subjected for incubation. On seventh day, a suitable window was opened and VEGF (50 ng) in ovalbumin coated coverslips of 12 mm diameter (with or without test substance) were placed over the chorio-allantoic membrane. Coverslips coated only with ovalbumin, ovalbumin with VEGF, ovalbumin with VEGF and thalidomide (10 μg), ovalbumin with VEGF and PHF (50 μl) served as normal, control, positive control and test respectively. A minimum of 8 eggs were used per group. The windows were further sealed with sterile parafilm and eggs were reincubated for further period up till twelfth day. On the day 12, the blood vessels grown under the coverslip area were digitally photographed using a sensitive CCD camera directly attached to the computer using graphic user interface. The blood vessel areas were analysed using Aphelion Developer Image Analysis Software (Adcis, France).

#### Corneal neovascularization assay

Corneal neovascularization was induced by chemical cauterization, using the technique described by Mahoney & Waterbury,1985 [5]. One cornea of each rat was cauterized by pressing an applicator stick (with a diameter of 1.5 mm) coated with 75% silver nitrate / 25% potassium nitrate on the centre of the cornea for 5 sec while the animal was deeply anesthetized with sodium pentobarbitone. To increase the reproducibility of the injuries, the same investigator cauterized all animals. Following cauterization, the rats were randomized to eliminate potential bias in the degree of injury within the different groups. Rats were randomly divided into three groups (n = 6) viz. sham, positive control (bevacizumab 1.25 mg/ml) and PHF treated. 20 μl of test was applied topically to each cauterized cornea three times per day for 5 days starting from the day of cauterization. The first treatment with each medication was started approximately 30 min after cauterization. The corneal neovascularization was assessed on day 6 after cauterization whereby the corneas of all rats were subjected for photography using CCD camera. The photographs were further subjected for quantification of corneal neovascularization using the Aphelion Dev. image analysis software (Adcis, France).

### Evaluation of PHF for its cytotoxicity

#### MTT assay

The HeLa cancer cell lines (No. 25) were obtained from National Centre for Cell Science (NCCS), Pune, India and suspended in 2.0 ml of DMEM (10% Fetal Bovine Serum). Cell count was adjusted to 5 × 10^4^ cells/ml. 100 μl of this cell suspension was plated in each well of 96-well ELISA plate. Plated cells were incubated in a CO_2_ incubator (5% CO_2_, 37°C) for 16 h, after which the media was removed from wells and fresh DMEM media containing 50 μl PHF was added in triplicates followed by further incubation for 24 h. After 24 h, DMEM media was removed and 100 μl of MTT (1 mg/ml) was added to each well followed by incubation for 3 h in CO_2_ incubator. After 3 h, media containing MTT was removed and 100 μl of DMSO was added to each well to dissolve the formazan crystals. Absorbance was taken at 570 nm and 655 nm.

### Evaluation of anticataract potential of PHF

#### Hydrocortisone induced cataract in developing chick embryos

The procedure previously standardized in our laboratory [6] was used for this experiment. Briefly, ten days old *White leghorn* chick eggs were procured from Kegg Farm (Gurgaon, Haryana, India). The eggs were randomly divided into three groups (n = 8) namely normal (saline treated), control and PHF treated respectively. All the eggs were incubated in a humidified incubator at 36 ± 2°C. On 15^th^ day, a hole was drilled in the eggshell over the air sac. Control and test groups received hydrocortisone (0.25 μM/0.1 ml) injected through the hole to induce cataract where as normal group received 0.1 ml of normal saline. The puncture was sealed with an adhesive tape and eggs were re-incubated. Control and test groups received 0.1 ml of normal saline and PHF at 3, 10 and 20 h after hydrocortisone injection respectively. After 48 h of the hydrocortisone injection the groups were sacrificed and the eyes were enucleated. The lenses were dissected out and were subjected for grading by two independent observers who were not aware of the treatment.

For the evaluation of lens, original grading of Nishigori *et al*., 1982 [7] was used, stage 1 - lens is clear and indistinguishable from normal (no cataract), stage 2 - faint opaque ring between the cortical region and nuclear region, stage 3 - distinct opaque ring between both the regions, stage 4 - lens has a pin hole size of clear area in opaque nucleus and stage 5 - opaque nucleus.

#### Selenite induced cataract model

Wistar female albino rats along with pups (6 days postnatal) were housed together. Nursing mother was fed with normal diet and water *ad libitum*. Two groups having mother and pups were divided. The pups of each rat were divided into two groups namely control and PHF treated and each group consisted of 6 pups respectively. The rat pups of the control and treated group were given sodium selenite at the dose of 25 μM/kg subcutaneously on the 9^th^ postnatal day. Treated group received PHF subcutaneously in the volume of 0.05 ml from the seventh postnatal day to sixteenth day. The eyes were subjected for torchlight and slit lamp examination on the sixteenth postnatal day and compared with control for the presence and absence of nuclear cataract [8].

#### *In vivo* galactose induced cataract in rats

Wistar albino rats of either sex weighing 80–100 g were fed with 30% galactose diet with water *ad libitum* for 28 days. The rats were divided equally into two groups of control and test, each consisting of 5 animals. Test group was instilled with PHF amounting to 20 μl three times a day and control animals received saline. Each eye was evaluated for the presence of cataract and was graded by Sippel’s method [9] using slit lamp on 7, 14, 21 and 28^th^ day.

##### Grading of lenses

Two independent observers those who were not aware of the treatment evaluated the stages of cataract using slit lamp biomicroscopy in rat eyes and mean of their grading was considered for further comparisons. The lenses were graded using the Sipple’s scale, Stage 1 - Clear lens, Stage 2 - Peripheral vacuoles, Stage 3 - Irregular peripheral vacuoles with the involvement of lens cortex, Stage 4 - Irregular opacity of lens and Stage 5 - Pronounced opacity.

### Evaluation of the PHF for its antioxidant activity

#### DPPH radical scavenging assay

The free radical scavenging activity of PHF was measured in term of hydrogen donating or radical scavenging ability using the stable radical DPPH [10]. Briefly, 1.0 ml of 0.1 mM solution of DPPH in ethanol was added to 3.0 ml of test solution (with varying dilutions) and 30 min later, the absorbance was measured at 517 nm. Sodium ascorbate was used as a positive control and deionized water served as control. Percentage radical scavenging activity (%RSA) was calculated by following equation:

$$\text{\%RSA}=\left(\mathrm{Ab}s_{\text{control}}-\mathrm{Ab}s_{\text{test}}\right)/\mathrm{Ab}s_{\text{control}}*100$$

Abs_control_ is the absorbance of control in presence of deionized water without test and Abs_test_ is the absorbance in presence of PHF or Sodium ascorbate. The antioxidant activity was expressed as IC_50_ (concentration (μg/ml) of the test that inhibited the formation of DPPH radicals by 50%).

### Evaluation of PHF for its anti-inflammatory activity

#### Carrageenan induced paw edema

Overnight fasted Wistar albino rats (n = 6) of either sex (100–150 g) were divided into control (saline treated), positive control (diclofenac 5 mg/kg bodyweight treated) and experimental (4 ml/kg PHF treated) groups. After one hour of intraperitoneal drug administration, paw edema was induced by single injection of 0.1 ml of 1% carrageenan injected intradermally on the plantar side of the right hind paw. Paw volumes were measured using a digital plethysmometer (Inco, Haryana) immediately before the injection of carrageenan and at hourly intervals for 4 h thereafter. The volume of edema was expressed for each rat as the difference before and after the injection of carrageenan. The percent inhibition of paw edema was calculated by following equation:

$$\text{\%Inhibition}=V_{c}–V_{t}/V_{c}*100$$

V_c_ is the paw volume of control that received saline and V_t_ is paw volume of test that received PHF or Diclofenac.

#### LeukotrieneB_4_ (LTB_4_) inhibition assay

Fresh buffy coat was obtained from hospital blood bank and subjected for WBC separation using HiSep (Hi-media, Mumbai) and diluted with equal volume of Hank’s balanced salt solution. The metabolism of endogenously bound arachidonic acid to LTB_4_ was measured in a total volume of 0.4 ml of the cell suspension at 37°C. The reaction tubes were containing 20 μg arachidonic acid and the investigational drugs (PHF and zileuton). After 45 min pre-incubation the reaction was started by addition of 20 μg of calcium ionophore and 2 μg of glutathione. After 15 min the reaction was stopped with 40 μl of 0.1 M HCl. After centrifugation for 2 min aliquots of the supernatant were subjected to LC-MS/MS estimation of LTB_4_.

Hypersil GOLD column (50 mm × 2.1 mm, 1.9 μm, Thermo, Waltham, MA, USA) with a gradient system of acetonitrile with 0.1% formic acid and water with 0.1% formic acid was used for chromatographic separation. The gradient was started with 80:20 water/acetonitrile and reached to 30:70 in 3 min and first line condition was achieved over a period of 2 min. Flow rate was maintained at 0.5 ml/min.

Tandem Mass spectrometric detection of analytes and internal standard (IS) was carried out with an electrospray ionization (ESI) source operated in the negative mode. Multiple Reaction Monitoring (MRM) mode was performed for quantification. 335.2 m/z was selected as precursor ion (Q1) whereas 317.2 m/z and 195.1 m/z (Q3) were selected as product ion for LTB_4_. Optimized compound dependent parameters were Declustering Potential (DP) - 76, Entrance Potential (EP) - 10, Collision Energy (CE) - 20 and Collision Cell Exit Potential (CXP) - 8. Probenecid was selected as an IS with 283.91 m/z (Q_1_) and 240 m/z (Q3).

### Evaluation of intraocular penetration of PHF

New Zealand Albino Rabbits (n = 4) of either sex weighing 2–2.5 kg body weight were used for this study. Briefly 50 μl of PHF was instilled after each 10 min for 1 h into the cul-de-sac of the rabbit eyes. After completion of dosing, eyes were thoroughly washed with normal saline and aqueous humor was aspirated at 2 h via paracentesis. Briefly, eyes were anaesthetized by instilling 4% lidocaine and 70–100 μl of aqueous humor was withdrawn with the help of 30 G sterile hypodermic needle. All the samples were stored at −70°C until analysis by LC-MS/MS.

For the detection of compounds in PHF, information dependent acquisition (IDA) method using a hybrid system of Ultra High Performance Liquid Chromatography (UHPLC) coupled with Mass Spectrometry (LC-MS/MS) was used.

UHPLC (Accela, Thermo Surveyor system, Thermo Electron Corp, Waltham, MA, USA) was equipped with a quaternary pump connected to an online degasser and photodiode array (PDA) coupled with triple quadrupole mass spectrometer (4000 Q-Trap, AB/MDS Sciex, Foster City, CA, USA). Luna® column (250 mm × 4.6 mm, 5 μm, Phenomenex, USA) with a linear gradient system of water with 0.1% formic acid and acetonitrile with 0.1% formic acid was used for chromatographic separation. Gradient was started with 10% organic mobile phase and reached to 100% with 18% increase in each 5 min.

Tandem Mass spectrometric detection was carried out on an Applied Bio Systems 4000 triple quadrapole instrument (ABS Biosystems, Foster City CA, USA) equipped with an ESI source that operated in the positive ion mode. For a mass range from 100 to 800 atomic mass unit (amu), IDA scanning was done at a speed of 1000 Da/s with Enhanced Mass Scan (EMS). Chromatogram for PHF obtained from IDA scan was screened for the masses available in literature.

The analytes which were found to be present in IDA scan for PHF were further confirmed by observing their fragmentation patterns using MRM mode in LC-MS/MS. For MRM, all compounds were optimized for their fragmentation patterns and compound dependent parameters and subsequent separation of compounds was achieved using Purospher® STAR RP-18 (55 mm × 4 mm, 3 μm, Merck, Darmstadt, Germany) column with guard. For chromatographic separation a linear gradient of water with 0.1% formic acid and acetonitrile with 0.1% formic acid was used in a starting ratio of 90% acetonitrile. The gradient reached to 100% organic phase with an increment of 1% in 1 min.

The aqueous humor samples were also subjected for the same optimized MRM conditions in LC-MS/MS. For this 50 μl of thawed aqueous humor samples were processed by adding 50 μl of extraction solvent [90% acetonitrile with 250 ng of sulfadimethoxine (IS)]. All the samples were then vortexed for 1 min and centrifuged at 3600 g for 10 min and further subjected for analysis.

### Statistical analysis

All the data are presented as mean ± SEM and statistical significance was calculated using Sigma plot version 11. For CAM and corneal neovascularization assay, groups were compared using unpaired student-t test. In carrageenan induced rat paw edema method different groups were compared using one way ANOVA followed by Tukey test.

## Results

### CAM assay

**Figure 1:**
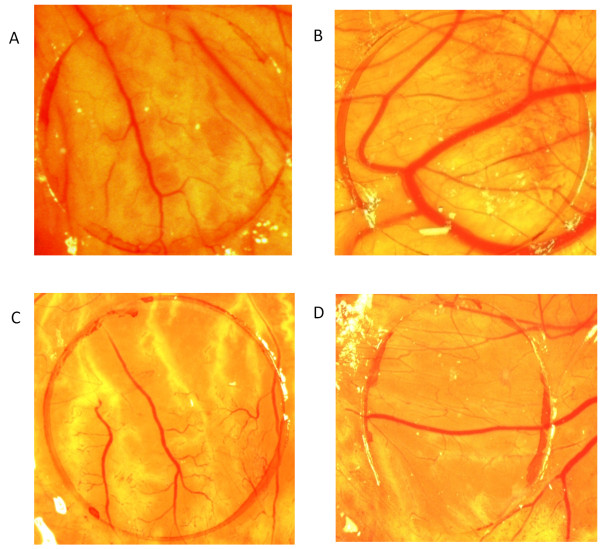
**Effect of PHF on the chick chorio-allantoic membrane observed on day 12 of incubation.** Photographs showing blood vessels on the Chorio-Allantoic Membrane (CAM) for different groups. **A**. Normal. **B**. Treated with VEGF 50 ng. **C**. Treated with Thalidomide 10 μg along with VEGF 50 ng. **D**. Treated with 50 μl of PHF along with VEGF 50 ng.

VEGF at a concentration of 50 ng was found to significantly induce the proliferation of new blood vessels in comparison to the normal group. PHF showed significant antiangiogenic activity (Figure 1) as it profoundly inhibited the VEGF induced proliferation of new blood vessels when compared to the VEGF control group (Table 1). Thalidomide was used as a positive control for the study whereby it was found to significantly inhibit the VEGF induced proliferation of new blood vessels at a concentration of 10 μg.

**Table 1:** Representation of vessel area per coverslip in CAM assay

**GROUP**	**VESSEL AREA / COVERSLIP (mm × mm)**
Normal	39.67 ± 0.38
Control (VEGF 50 ng)	90.35 ± 0.33
Thalidomide 10 μg + VEGF 50 ng	27.65 ± 0.31
PHF 50 μl + VEGF 50 ng	48.06 ± 0.4*

Data are presented as mean ± SEM (n = 8). *p ≤ 0.001 vs Control Group.

### Cautery induced corneal neovascularization

**Figure 2:**
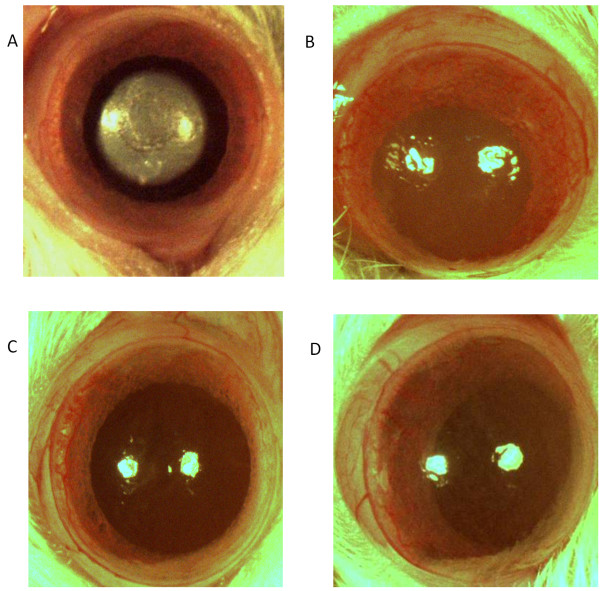
**Effect of PHF in cautery induced corneal neovascularization in rats. **Photographs showing rat corneas for different groups. **A**. Cauterized cornea. **B**. Sham treated cornea. **C**. Bevacizumab 1.25 mg/ml treated cornea. **D**. PHF 50 μl treated cornea.

There was no corneal neovascularization observed in the normal (uncauterized) corneas. The PHF group showed significant antiangiogenic activity in the cautery induced corneal neovascularization assay (Figure 2) in comparison to the sham treated group which showed considerable neovascularization in the cornea of rat eyes (Table 2). The positive control (bevacizumab) was observed to significantly inhibit the cautery induced corneal neovascularization at a concentration of 1.25 mg/ml.

**Table 2:** Vessel area in the cauterized corneas of different groups

**GROUP**	**AREA OF CORNEAL BLOOD VESSELS (mm × mm)**
Sham	15.37 ± 0.5
Bevacizumab 1.25 mg/ml (20 μl)	4.95 ± 0.22
PHF (20 μl)	12.48 ± 0.37*

Data presented as Mean ± SEM (n = 6). *p ≤ 0.001 vs Sham treatment.

### Cytotoxicity

The LD_50_ for taxol was found to be 5.05 μg/ml whereas the PHF did not exhibit any cytotoxicity against the HeLa cancer cell lines.

### Hydrocortisone induced chick cataract

**Figure 3:**
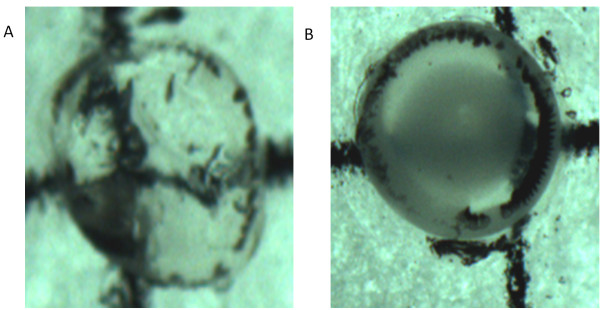
**The effect of PHF on lenses in the hydrocortisone induced cataract model in chicks.** Photographs showing lenses in different stages of cataract. **A**: Stage 1: Normal group **B**: Stage 4: PHF treated group.

The normal (untreated by hydrocortisone) group evinced no development of cataract and control group was graded for stage V (nuclear opacity). In this model PHF showed only a slight protection against cataract as compared to the control group and was graded for stage IV cataract (Figure 3).

### *In vivo* selenite induced oxidative model of cataract

The PHF treated group showed significant protection as compared to the control group. In the control group, 100% eyes showed cataract development and were graded for stage IV. Whereas, the eyes treated with PHF showed noticeable delay in the progression of cataract and were graded for stage II. The observations clearly demonstrated the potential of the PHF in delaying the progression of cataract in comparison to the control group.

### *In vivo* galactose induced cataract in rats

**Figure 4:**
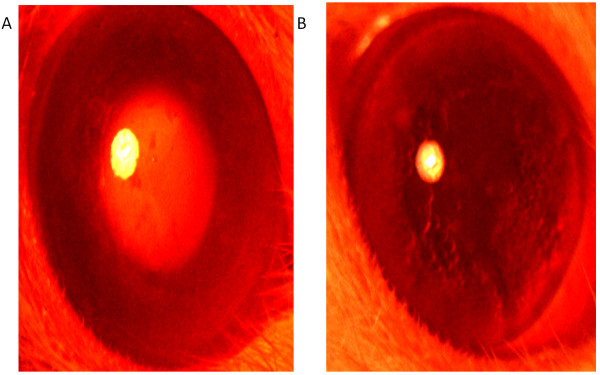
**The effect of polyherbal formulation on lenses in the galactose induced cataract model in rats. **Photographs showing lenses in different stages of cataract: **A**: Stage 5: Control group **B**: Stage 3: PHF group.

At the end of 4 weeks (after galactose feeding) the control group showed significant development of cataract. The lenses in control group depicted stages V respectively, as per Sipple’s gradings. The PHF treated lenses showed slight delay in the development of cataract and were graded for stage III (Figure 4).

### DPPH radical scavenging assay

The IC_50_ of the standard (sodium ascorbate) was found to be 5.9 μg/ml. comparatively, the PHF did not show any significant antioxidant activity and the percentage radical scavenging activity up to the maximum of 1.25 ml of PHF was found to be only 26.6%.

### Carrageenan induced paw edema

Diclofenac showed maximum inhibition of paw edema (63.6%) after 3 h of its induction via carrageenan. Comparatively, the PHF showed maximum inhibition of paw edema (51.8%) after 3 h (Table 3).

**Table 3:** Effect of PHF and diclofenac on carrageenan induced paw edema in rats (after 3 hr)

**GROUP**	**PAW VOLUME (ml)**	**PERCENTAGE INHIBITION (%)**
Control	1.1 ± 0.02	-
Diclofenac (5 mg/kg body weight)	0.4 ± 0.02^a^	63.63
PHF (4 ml/kg body weight)	0.53 ± 0.02^a^	51.8

Data are presented as mean ± SEM (n = 6). ^a^P ≤ 0.001 when compared with control.

### *In vitro* LTB_4_ inhibition assay

**Figure 5:**
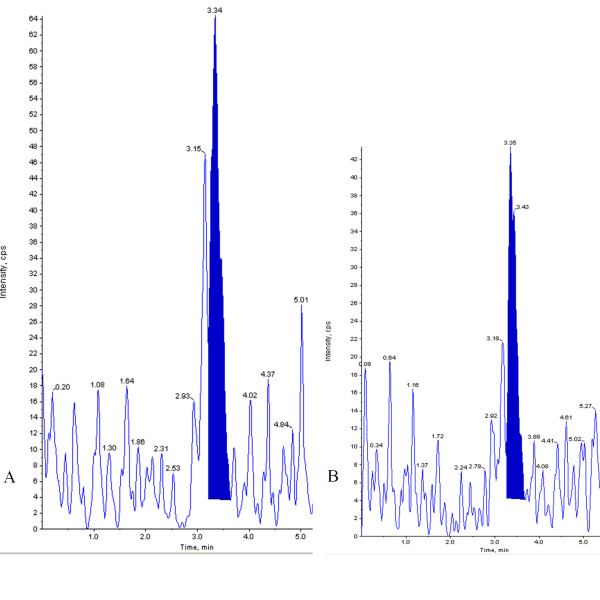
**LC-MS/MS chromatograms showing levels of LTB**_**4 **_**in human WBC’s. A**: Control group, **B**: PHF treated.

In the *in vitro* method involving LTB_4_ inhibition from human WBC, zileuton was used as a positive control that showed 100% inhibition of LTB_4_ formation at the tested concentration of 20 μg as compared to untreated control. Among various volumes tried, test group received 20 μl of PHF showed 40% inhibition of LTB_4_ formation as compared to the control group (Figure 5).

### Intraocular penetration of PHF

PHF tested by IDA protocol showed presence of ar- turmerone, camphor, casticin, curcumin-I, curcumin-II, iso-orientin, luteolin, menthol, quercetin, rosmarinic acid, thymol, wedelolactone, sabinene, γ-terpinene, β- pinene, dipentene. These compounds were further confirmed by MRM.

**Figure 6:**
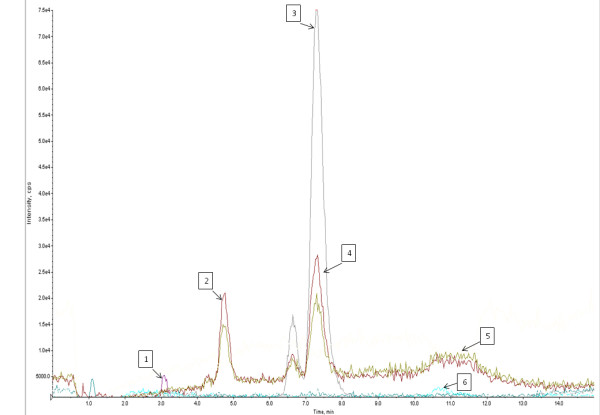
**LC-MS/MS chromatogram of aqueous humor sample showing intraocular penetration of PHF in rabbit eyes. **MRM chromatogram of compounds with high intensity peaks: **(1)** Quercetin, **(2) **γ- terpinene, **(3) **Rosmarinic acid, **(4) **β-pinene, **(5) **Dipentene, **(6) **Curcumin II.

Aqueous humor subjected for optimized MRM for PHF showed presence of camphor, casticin, curcumin-II, quercetin, rosmarinic acid, γ-terpinene, β-pinene and dipentene (Figure 6).

## Discussion

The polyherbal eye drop (Itone™) is a sterile topical formulation that has been found to have pH 5.1 and is hypo-osmolar in nature. It has been claimed to protect eyes from continuous strains, glares of light and various forms of pollution upon its instillation. It has also been indicated as an adjunct in various ophthalmic conditions like conjunctivitis, trachoma, blepharitis, keratitis, corneal ulcers, lentricular opacity, myopia, hypermetropia and dacrocystitis [11]. Experiments so far evaluated using PHF showed several properties viz., lack of any acute, subacute CNS toxicity after oral administration in animals, lack of ocular irritancy in rabbits and prominent antimicrobial activity against *Staphylococcus aureus* and *Klebsiella pneumoniae*[11]. In clinical studies, the PHF under study has been found to be effective in trachoma, chronic conjunctivitis [12], subjective improvement in refractory errors [13] healing capacity in allergic conjunctivitis [13], viral conjunctivitis [14,15] and computer vision syndrome [2]. Although, enough literature is available regarding the above therapeutic uses for the PHF, no systematic study is available to substantiate the claim using controlled experiments. Therefore, the present study was conducted to evaluate antiangiogenic, anticataract, anti-inflammatory, antioxidant and cytotoxic potential of the PHF using several experimental models. As PHF under study was also reported to provide relief from iridocyclitis, early senile lenticular opacity and IOP lowering effect in glaucoma, its intraocular penetration was felt as necessary. Therefore, this study was extended to evaluate the intraocular penetration in the rabbit eyes upon topical application using liquid chromatography coupled tandem mass spectrometry.

Ocular angiogenesis is responsible for the majority of irreversible blindness in the developed world. This debilitating complication affects all age groups and characterizes such diverse and widespread diseases as trachoma, retinopathy of prematurity, diabetic retinopathy, neovascular glaucoma and age-related macular degeneration [16]. Angiogenesis is a tightly regulated process involving the development of new blood vessels from pre-existing blood vessels. During development and normal physiological processes such as wound healing and the menstrual cycle, angiogenesis is regulated by endogenous activators and inhibitors [17,18]. In pathological settings, such as age-related macular degeneration, rheumatoid arthritis, diabetic retinopathy and tumor growth and metastasis, angiogenesis is critical for disease progression [17,19].

The present study evaluated the antiangiogenic potential of a PHF using the *in ovo* chick CAM assay – an assay that is capable of evaluating the action of test substance (plant extracts) on angiogenesis [20]. For this assay, VEGF 50 ng was used as an angiogenesis stimulator and was found to significantly induce proliferation of new blood vessels as compared to the normal group. In this assay, PHF was found to have significant antiangiogenic potential at the studied concentration thereby inhibiting VEGF induced proliferation of new blood vessels.

In order to understand the *in vivo* significane of the above finding an animal study was carried out using chemical cautery induced corneal neovascularization in rats. Neovascularization has been reported as an important pathologic event during the corneal wound healing process [21] and VEGF has been reported to play an important role in its pathogenesis [22]. Neovascularization of cornea may cause loss of corneal transperancy and thereby leads to loss of vision [23]. In the present study, topical instillation of the PHF was found to significantly inhibit chemical cautery induced corneal neovascularization as compared to sham treated control. In this assay, bevacizumab, a known anti-VEGF monoclonal antibody was used as a positive control that showed 67% inhibition of the cautery induced corneal neovascularization in comparison to the sham treated group that showed noticeable corneal neovascularization in the cauterized eyes. The antiangiogenic activity observed for PHF was found to be only 18%, thereby concluding that the antiangiogenic activity observed with PHF is mild in comparison to the potential antiangiogenic compounds like bevacizumab.

Interestingly, the present study revealed that the PHF was found to be safe upon application to the chick embryos and there was no toxicity found. Chorio-allantoic membrane assay is also a well recognized method to study ocular toxicity of drugs [24]. It is assumed as acute irritating effects on the small blood vessels and protein membrane are similar to effect induced by same chemical in the eye [25]. Further to the above observation, lack of any cytotoxicity of the studied PHF was confirmed in HeLa cell lines using MTT assay. In this cytotoxicity assay paclitaxel was used as positive control. This study further confirms lack of any ocular toxicity oberved in majority of the above studies [11].

Inflammatory stimulus is a factor responsible for micro-vascular gowth (neovascularization) during the active pro-angiogenic phase. Therefore, capillary regression is of interest from a clinical perspective [26]. Therapeutic strateties for reducing the vacular growth has been well accepted as a therapeutic option for pathologic angiogenesis in tumors, eye diseases, and inflammation. Anti-inflammatory compounds like COX-2 inhibitors and steroids are reported to have anti-angiogenic potential [27,28]. Therefore, the present study was extended to carry out anti-inflammatory property of the PHF in the carageenan induced paw edema model of inflammation.

Inflammation is a normal protective responce to tissue injury caused by physical trauma, noxious chemical or microbial agents and involves release of various inflammatory mediators such as leukotrienes and prostaglandins [29-31]. In the present study, anti-inflammatory activity of PHF was tested using carrageenan induced paw edema assay which has been used since time as a standard technique for the screening of anti-inflammatory activity of several herbal extracts [32,33]. PHF was observed to possess noticeable anti-inflammatory activity, though the activity was comparatively lesser than the positive control (diclofenac). Furthermore, PHF was evaluated for its inhibitory effect on the formation of LTB_4_ in human WBCs. LTB_4_ is a known potent inflammatory mediator and has been implicated as a probable cause of chronic ocular inflammation and retinopathy in diabetes [34] and has also been held responsible for the development and progression of experimental autoimmune uveitis [35]. Our study showed that PHF inhibited the formation of LTB_4_ in human WBCs while the inhibitory percentage was relatively lower in comparison to zileuton which is a known lipoxygenase inhibitor.

The widespread prevalence of diabetes in developing countries is expected to increase the magnitude of blindness due to cataract and the cellular and molecular mechanisms underlying the pathogenesis of cataract showed the involvement of polyol pathway, advanced glycation end products and oxidative stress [36]. The therapeutic agents that are capable of altering the above events are expected to prevent or delay the progression of cataract in order to exhibit the desired anti-cataract activity e.g. chyavanprash [37]. Considering the major pathways involved in the etiology of cataract such as polyol pathway or sorbitol accumulation pathway, anticataract potential of PHF was evaluated using various pharmacological screening models mimicking the major pathways in cataract. In the developing chick embryo model of cataract, glucocorticoid (hydrocortisone) produced higher incidence (>90%) cataractous changes in lenses within 48 h [38]. Cataract formation is caused by oxidative stresses, probably derived from hydrocortisone effects on the main target organ, the liver and can be prevented by radical scavengers including ascorbic acid and insulin. In this model of hyperglycemia and oxidative changes in lens including glutathione depletion are emphasized as factors for the loss of transparency. The present study observed a delay in the progression of cataract in all three models of cataract as compared to their untreated controls. However, the PHF did not show any noticeable free radical scavenging activity in the DPPH assay thereby demarking the presence of any antioxidant potential at the studied concentration. From this observation, it is evident that the mild anticataract activity observed with PHF could be beyond its antioxidant property or as a cumulative effect of antioxidant compounds after repeated administration.

In order to evaluate the possible intraocular penetration of PHF, a study was conducted by taking the aqueous humor after multiple topical instillations for availability of compounds present in PHF. Information dependent acquision method was develpoed and used for the testing of the availability of compounds in PHF. Furthermore presence of compounds in PHF was confirmed by MRM and used for aqueous humor analysis. This study proved that some of the compounds found in PHF have the propansity to cross the cornea to produce the intraocular effects. Among the penetrated compounds curcumin, rosmarinic acid and quercetin are capable of having anticataract activity as reported previously [39-41] whereby curcumin has also been reported for its antiangiogenic potential [42]. However, further studies with isolated and purified compounds are essential to reveal the extent of their penetration for their correlation with observed activity. As the PHF is a proprietary Ayurvedic formulation having many components with very low concentrations, the mild but significant protective effects observed on inflammation, angiogenesis and cataract can have cumulative and synergestic effects while on repeated topical applications in patients for their obvious therapeutic effect with low or no toxicity as observed in clinical studies.

## Conclusions

The PHF showed significant antiangiogenic and anti-inflammatory activities and noticeable anticataract activity. However, the PHF did not exhibit any antioxidant potenial and did not show any cytotoxicity against HeLa cancer cell lines. Moreover, the intraocular penetration studies revealed that some of the components of PHF were capable of having transcorneal penetration. The significant antiangiogenic and anti-inflammatory activities evinced by the PHF merits further investigation of PHF for ocular neovascular and inflammatory diseases in humans.

## Competing interests

Dey’s Medical Store, Kolkata is the manufacturer of Itone™ who has provided preservative free formulation and academic research grant for conducting this scientific study. The authors hereby declare that the manufacturer had no role or interest in the design, conduct and interpretation of the study.

## Authors’ contributions

VT designed the experiments and drafted the manuscript. GP carried out the pharmacological screening of PHF. RAK carried out the anticataract screening of PHF. SHP carried out intraocular penetration of PHF and anti-inflammatory screening using carrageenan induced paw edema assay. BNR participated in the design and co-ordination of the study. All the authors read and approved the final manuscript.

## Pre-publication history

The pre-publication history for this paper can be accessed here:

http://www.biomedcentral.com/1472-6882/13/1/prepub
